# Complete genome sequence of Japanese encephalitis virus strain SDWF-2021 isolated from a *Culex* mosquito pool from a duck farm

**DOI:** 10.1128/mra.00841-23

**Published:** 2023-12-04

**Authors:** Chenxi Li, Wen Zhao

**Affiliations:** 1 College of Veterinary Medicine, Yangzhou University, Yangzhou, China; 2 State Key Laboratory of Veterinary Biotechnology, Harbin Veterinary Research Institute of the Chinese Academy of Agricultural Sciences, Harbin, China; 3 Jiangsu Co-Innovation Center for Prevention and Control of Important Animal Infectious Diseases and Zoonoses, Yangzhou, China; 4 College of Animal Science and Technology, Yangzhou University, Yangzhou, China; 5 Department of Agricultural and Animal Husbandry Engineering, Cangzhou Technical College, Cangzhou, China; Katholieke Universiteit Leuven, Leuven, Belgium

**Keywords:** Japanese encephalitis virus, genotype I

## Abstract

We report here the complete genome sequence of Japanese encephalitis virus (JEV) strain SDWF-2021, isolated from a *Culex* mosquito pool in a duck farm located in Shandong, China. The isolated JEV genetically belong to genotype I, which is the dominant genotype circulation in China.

## ANNOUNCEMENT

Japanese encephalitis virus (JEV), belonging to genus *Flavivirus* of the family Flaviviridae, is an enveloped, positive-sense single-stranded RNA virus (1). JEV is an important zoonotic pathogen with a transmission cycle maintained by mosquito vectors (*Culex* mosquitoes) and vertebrate-amplifying hosts (birds and pigs) (2, 3), and it causes encephalitis in humans and abortion and orchitis in breeding pigs (4, 5). JEV is phylogenetically divided into five genotypes (GI to V) based on the nucleotide sequence of the E gene (2).

In July 2021, approximately 1,000 *Culex* mosquitoes were collected from a duck farm located in Hanting District, Weifang city of Shandong Province in China. Mosquitoes were pooled per 20, grounded in RNase-free water, and centrifuged at 4°C to collect the supernatant. RNAs in supernatant were extracted by the TIANamp Virus RNA Kit (TIANGEN, Beijing, China) and utilized for the detection of JEV by an established duplex TaqMan probe-based RT-qPCR assay (6). A total of 1/51 (1.96%) *Culex* mosquito pools gave positive result. Subsequently, the supernatant of JEV-positive sample was collected to seed onto monolayers of BHK-21 cells and incubated at 37°C, 5% CO_2_ for 3 days. The supernatant of cells with typical cytopathic effects was collected to pass in BHK-21 cells up to five times. RNA was extracted from the fifth passage and reverse transcribed into cDNA using the Superscript IV first-strand synthesis system (ThermoFisher Scientific, Waltham, MA, USA), and four pairs of primers targeting conserved regions of the full-length sequences of GI and GIII JEV were designed to amplify the complete genome of SDWF-2021 (2). Meanwhile, the 5′ and 3′ terminal ends of viral genome were amplified using the SMART 5′ RACE and 3′ RACE (Takara, Kyoto, Japan) and a gene-specific primer (Table 1). A total of four overlapping fragments (F1, F2, F3, and F4) were amplified and cloned into the pEasy-blunt vector (TransGen Biotech, Beijing, China). Three or four clones from each PCR product were sequenced via Sanger’s DNA sequencing using the primers based on conserved regions of the full-length sequences of GI JEV (Table 1). The sequences of four fragments were assembled into the complete genome of JEV SDWF-2021 strain, and the open reading frame was determined by comparison with the classical strain SA-14 (GenBank accession number KU323483.1).

**TABLE 1:** Primers used in this study

Primer	Primer sequence (5′ to 3′)	Target nucleotide positions	Application
F1-F^*a*^	agaagtttatctgtgtgaact	1–21	Amplification and sequencing of F1 fragments
F1-R^*a*^	ttgtgtgatccaagacattcccccaaagag	2331–2361	
F2-F	ctctttgggggaatgtcttggatcacacaa	2331–2361	Amplification and sequencing of F2 fragments
F2-R	tggaacaccgggatcatcaatcaagtgaaa	4467–4497	
F3-F	tttcacttgattgatgatcccggtgttcca	4467–4497	Amplification and sequencing of F3 fragment
F3-R	attatgccagccgctgttcttctctga	7322–7349	
F4-F	tcagagaagaacagcggctggcataat	7322–7349	Amplification and sequencing of F4 fragment
F4-R	ctggtggtgaggaagaacacaggatct	10939–10965	
S1-R	tgttggtttgtcgtttgccataattgtcaa	1068–1098	5′ RACE and sequencing of F1 fragment
S2-F	ttcatagaaggagccagtggagccacttgg	1008–1038	Sequencing of F1 fragment
S3-F	gcgttggcaggagccatcgtggtggagtac	1770–1800	
S4-F	cccgagtgtggctgaaaattagag	2971–2995	Sequencing of F2 fragment
S5-F	cttgtgctgatgcttgggggcatcacttac	3657–3687	
S6-F	ataggaatttgttccctgctgcaaga	4062–4088	
S7-R	ccggttttgtctggatgtttactgc	4923–4948	Sequencing of F3 fragment
S8-F	gcaatgtgcctccaaagagcggggaaaaaggtcatcc	5724–5761	
S9-R	ggatgacctttttccccgctctttggaggcacattgc	5724–5761	
S10-F	tgggcggcagaggttcctggaaccaaaatagc	6747–6779	
S11-R	gctattttggttccaggaacctctgccgccca	6747–6779	
S12-F	ggaagtgctcaacgagaccaccaactggct	8795–8825	Sequencing of F4 fragment
S13-R	agccagttggtggtctcgttgagcacttcc	8795–8825	
S14-F	ggtcattggaccacaacacttggaacagct	9566–9596	3′ RACE and sequencing of F4 fragment

^*a*^ F refers to forward primer, and R refers to reverse primer.

The complete genome sequence of SDWF-2021 was 10,965 bp in length, and its G + C content was 51.72%. Based on the processing principles of flavivirus polyprotein (7), the polyprotein of JEV SDWF-2021 strain was composed of 10 proteins, including 3 structural proteins (C, prM, and E) and 7 nonstructural proteins (NS1, NS2A, NS2B, NS3, NS4A, NS4B, and NS5). Nucleotide similarity analysis indicated that the complete genome sequence of SDWF-2021 displayed the nucleotide sequence identities of 88.8% and 89.1% to vaccine strain SA-14-14-2 (GenBank accession number JN604986.1) and classical strain SA-14, respectively.

**Fig 1:**
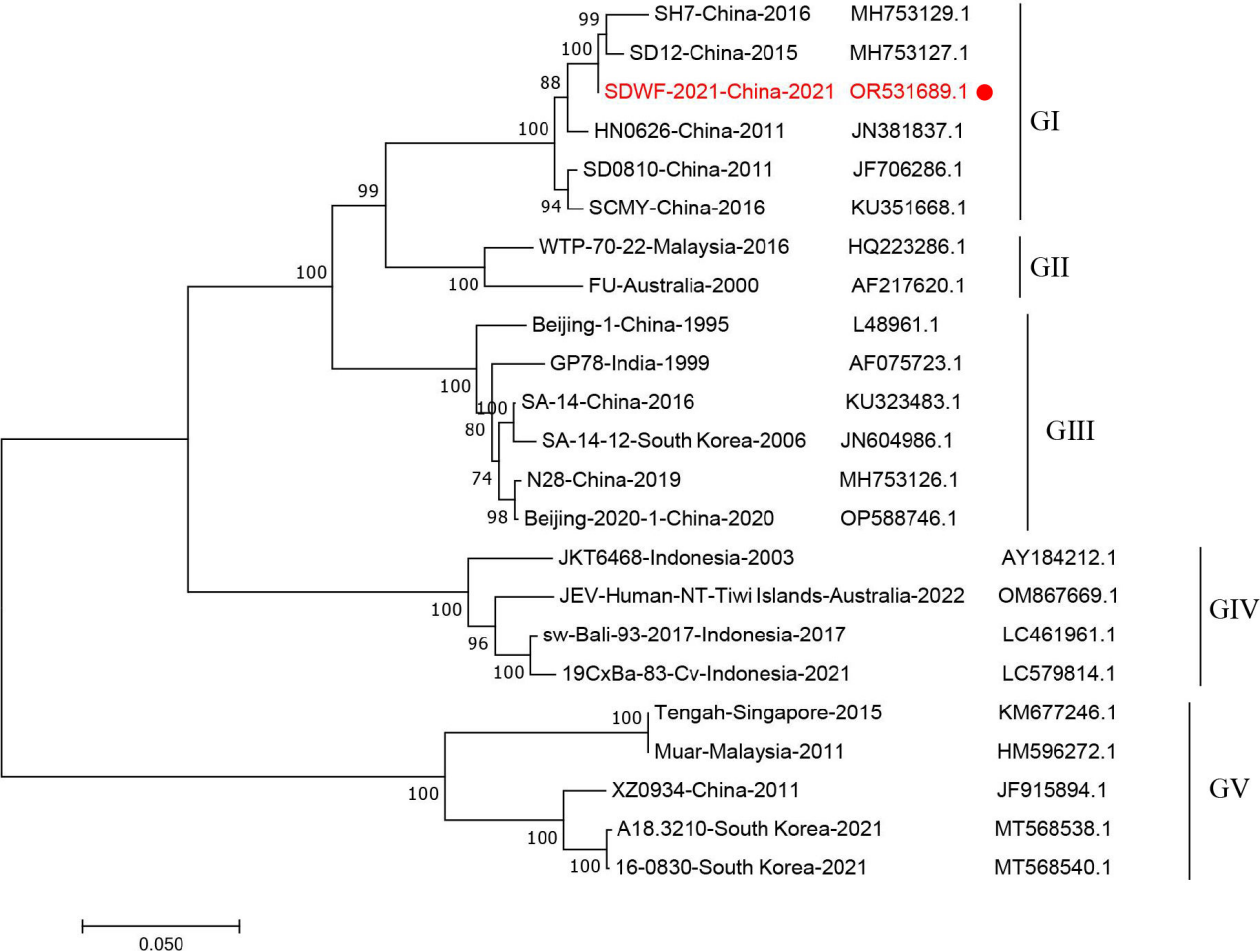
Phylogenetic analysis of JEVs based on nucleotide sequences of E gene. A total of 23 JEV strains including the strain isolated in this study (covering five genotypes), with clear background origin and known full genome sequences, were selected for phylogenetic analysis. The phylogenetic tree was constructed by the neighbor-joining method in MEGA 7.0 software (Kimura two-parameter model; 1,000 bootstrap replicates), yielding only values greater than 70%. The tree is drawn to scale, with branch lengths measured in the number of nucleotide substitutions per site. Five distinct sublineages were identified: GI, GII, GIII, GIV, and GV. Red dot indicates the JEV isolate (SDWF-2021) from the *Culex* mosquito pools.

Phylogenetic comparison of the E gene sequence of SDWE-2021 to those of other JEV strains (Fig. 1) showed that SDWE-2021 belongs to GI, which is the dominant genotype circulation in China (6). To date, the data on the prevalence of GI JEV in mosquitoes are lacking in China. The sequences of JEV SDWF-2021 strain will facilitate future research on the epidemiology and evolutionary biology of GI JEV in China.

## Data Availability

The complete genome sequence of JEV SDWF-2021 strain has been deposited in GenBank under the accession number OR531689.1. The BioProject accession number PRJNA1013244.
